# Auditing HIV Testing Rates across Europe: Results from the HIDES 2 Study

**DOI:** 10.1371/journal.pone.0140845

**Published:** 2015-11-11

**Authors:** D. Raben, A. Mocroft, M. Rayment, V. M. Mitsura, V. Hadziosmanovic, Z. M. Sthoeger, A. Palfreeman, S. Morris, G. Kutsyna, A. Vassilenko, J. Minton, C. Necsoi, V. P. Estrada, A. Grzeszczuk, V. Svedhem Johansson, J. Begovac, E. L. C. Ong, A. Cabié, F. Ajana, B. M. Celesia, F. Maltez, M. Kitchen, L. Comi, U. B. Dragsted, N. Clumeck, J. Gatell, B. Gazzard, A. d’Arminio Monforte, J. Rockstroh, Y. Yazdanpanah, K. Champenois, M. L. Jakobsen, A. Sullivan, J. D. Lundgren, HIDES Audit Study Group

**Affiliations:** 1 CHIP, Rigshospitalet, Copenhagen, Denmark; 2 University College London, London, United Kingdom; 3 Chelsea and Westminster Hospital NHS Foundation Trust, London, United Kingdom; 4 Gomel State Medical University, Gomel, Belarus; 5 Clinical Center University of Sarajevo, Infectious Diseases Clinic, Sarajevo, Bosnia; 6 Ben Ari Institute of Clinical Immunology, Rehovot, Israel; 7 University Hospitals of Leicester NHS Trust, Leicester, United Kingdom; 8 Western General Hospital, Edinburgh, United Kingdom; 9 Luhansk AIDS Center, Luhansk, Ukraine; 10 Belarusian State Medical University, Minsk, Belarus; 11 St James’s University Hospital, Leeds, United Kingdom; 12 Saint-Pierre University Hospital, Brussels, Belgium; 13 Hospital Universitario San Carlos, Madrid, Spain; 14 Medical University of Bialystok, Department of Infectious Diseases and Hepatology, Bialystok, Poland; 15 Department of Infectious Diseases, Karolinska University Hospital, Stockholm, Sweden; 16 University Hospital of Infectious Diseases, Zagreb, Croatia; 17 The Newcastle upon Tyne Hospital, Newcastle, United Kingdom; 18 Centre Hospitalier Universitaire de Fort de France, Fort de France, Martinique; 19 Centre Hospitalier de Tourcoing, Tourcoing, France; 20 Unit of Infectious Diseases University of Catania, ARNAS Garibaldi, Catania, Italy; 21 Hospital Curry Cabral, Lisbon, Portugal; 22 Medical University of Innsbruck Innsbruck, Austria; 23 Unit of Infectious Diseases, San Paolo Hospital, Milan, Italy; 24 Roskilde Hospital, Roskilde, Denmark; 25 Hospital Clinic de Barcelona, Barcelona, Spain; 26 University of Bonn, Bonn, Germany; 27 IAME, UMR 1137, Univ Paris Diderot, Sorbonne Paris Cité, Paris, France; 28 IAME, UMR 1137, INSERM, Paris, France; 29 AP-HP, Hôpital Bichat, Service de Biostatistique, Paris, France; Alberta Provincial Laboratory for Public Health/ University of Alberta, CANADA

## Abstract

European guidelines recommend the routine offer of an HIV test in patients with a number of AIDS-defining and non-AIDS conditions believed to share an association with HIV; so called indicator conditions (IC). Adherence with this guidance across Europe is not known. We audited HIV testing behaviour in patients accessing care for a number of ICs. Participating centres reviewed the case notes of either 100 patients or of all consecutive patients in one year, presenting for each of the following ICs: tuberculosis, non-Hodgkins lymphoma, anal and cervical cancer, hepatitis B and C and oesophageal candidiasis. Observed HIV-positive rates were applied by region and IC to estimate the number of HIV diagnoses potentially missed. Outcomes examined were: HIV test rate (% of total patients with IC), HIV test accepted (% of tests performed/% of tests offered) and new HIV diagnosis rate (%). There were 49 audits from 23 centres, representing 7037 patients. The median test rate across audits was 72% (IQR 32–97), lowest in Northern Europe (median 44%, IQR 22–68%) and highest in Eastern Europe (median 99%, IQR 86–100). Uptake of testing was close to 100% in all regions. The median HIV+ rate was 0.9% (IQR 0.0–4.9), with 29 audits (60.4%) having an HIV+ rate >0.1%. After adjustment, there were no differences between regions of Europe in the proportion with >0.1% testing positive (global p = 0.14). A total of 113 patients tested HIV+. Applying the observed rates of testing HIV+ within individual ICs and regions to all persons presenting with an IC suggested that 105 diagnoses were potentially missed. Testing rates in well-established HIV ICs remained low across Europe, despite high prevalence rates, reflecting missed opportunities for earlier HIV diagnosis and care. Significant numbers may have had an opportunity for HIV diagnosis if all persons included in IC audits had been tested.

## Introduction

Late-stage diagnosis of HIV and undiagnosed HIV continue to be features of many European HIV epidemics [1]. Despite extensive work and the widespread use of a consensus definition of late presentation, 50% of patients newly diagnosed with HIV have a CD4 count <350 cells/uL at diagnosis, negatively impacting both individual and public health [1–4]. Innovative approaches to better target testing for those most likely to be infected with HIV and who present late for care need to be developed. Studies suggest HIV testing will be cost-effective if the detected HIV prevalence in such testing programmes exceeds 0.1% [5–8].

As described elsewhere [9], the pilot phase of the HIDES Study (HIV Indicator Diseases across Europe Study) surveyed eight indicator conditions (ICs)–with 3588 individuals presenting with either sexually transmitted infections, malignant lymphoma, anal or cervical cancer, herpes zoster, hepatitis B and C, ongoing mononucleosis-like illness, unexplained leukocytopenia and thrombocytopenia and seborrheic dermatitis offered an HIV test. Out of the 3588 individuals, 66 were diagnosed with HIV. All eight ICs individually fulfilled the study’s criteria of demonstrating an HIV prevalence of >0.1%, however, for malignant lymphoma and anal and cervical cancer, 0.1 fell within the 95% confidence interval. [9]. In line with ECDC (European Centre for Disease Prevention and Control) guidance, the study demonstrated that individuals presenting to any healthcare setting with HIV indicator conditions (ICs) should be strongly recommended to have an HIV test [9–11].

Guidance for implementing HIV testing in adults in healthcare settings has been developed by HIV in Europe and widely disseminated. A number of countries have translated the document or included the recommendations in national testing guidelines/ recommendations [11]. The guidance divides HIV indicator conditions into three categories: 1) conditions which are AIDS defining among people living with HIV (PLHIV); 2) conditions associated with an undiagnosed HIV prevalence of >0.1% and 3) conditions where not identifying the presence of HIV infection may have significant adverse implications for the individual’s clinical management.

The present follow-up study, HIDES II, is expanding this testing strategy by increasing the number of indicator conditions and centres involved to identify those ICs with an HIV prevalence of >0.1%, [8, 9], and to ascertain whether there is variation in prevalence across Europe.

Adherence to testing guidelines across Europe in the context of IC-guided testing is currently unknown. Therefore, a second objective of HIDES II is to implement and evaluate an audit system across Europe of HIV testing of persons presenting with ICs where an HIV test should already be offered according to contemporary HIV testing guidelines [10,11]. As the pilot phase of HIDES identified a number of barriers to introducing IC-guided testing in “new” ICs, the audits aimed to investigate whether HIV testing is more routinely offered in already established ICs, and if regional differences exist.

## Methods

Six HIV indicator conditions were selected for auditing of HIV testing: tuberculosis (TB), hepatitis B and C (HEP), non-Hodgkin lymphoma (NHL), anal and cervical cancer (ACCAN) and oesophageal candidiasis (ECAN). These conditions fall into the category of AIDS defining conditions and/or conditions where the need for an HIV test is already widely accepted and should be part of clinical practice according to European and national HIV testing guidelines [10,11].

Each audit assessed the HIV test rate for one specific indicator condition for a specific segment of the population within a specific setting. It included all consecutive patients > 18 and <65 years of age, not known to be HIV positive, who had presented at the centre within the previous year or the last 100 consecutive patients or more seen at the centre. Participating centres reviewed retrospectively how many patients presenting with the IC were tested for HIV. Where data was available, information on the number of HIV tests offered and accepted (offer and uptake rates) was recorded. Each participating centre could complete one audit per IC.

A call for collaboration was sent to healthcare centres/hospitals across the four regions of Europe: North, East, South and West [12]. Centres were eligible for participation if they routinely saw patients with one or more of the six ICs and were selected based on an aim of delivering a balanced number of audits per IC and regionally within the study.

Data was collected retrospectively from May 2013. Centers reviewed medical reports and submitted data electronically to the coordinating centre via an online CRF system (REDCap) [13]. The offer rate was defined as number offered an HIV test divided by the number of patients seen with unknown HIV status. The uptake rate was defined as the number tested for HIV divided by the number offered a test. The test rate was defined as the number tested divided by the number of patients seen with unknown HIV status and the HIV+ rate was defined as the number testing HIV+ divided by the number of patients seen with unknown HIV status. A high test rate was defined as a test rate above the median of 72% and a high offer rate as a offer rate above the median of 86%.

All rates were standardised for duration of the audit and number of patients seen within a calendar year. Data are reported as medians and interquartile ranges (IQR) across rates, as individual patient data was not available.

ICs were combined to non-malignant AIDS (TB and oesophageal candidiasis), malignant (NHL, anal and cervical cancer) and hepatitis to allow comparison across ICs. Regions were defined according to regions defined in EuroSIDA [12].

As centres were not independent (i.e. more than one audit originated from a number of centres), generalised estimating equations with a binomial distribution was used to determine odds of outcomes, using robust standard errors to account for repeated audits within centres. Two primary outcomes were investigated: the odds of having a high test rate, defined as greater than the median test rate across all audits, and a high testing positive rate, defined as >0.1% testing positive, the cut-off used to determine economic viability [7,8].

As an additional exercise, observed HIV positivity rates were applied for each region and IC to estimate the number of HIV diagnoses potentially missed. The HIV+ rate and 95% confidence interval was applied within each region and IC to all patients seen over the relevant time period to estimate the number of HIV positive diagnoses potentially missed, using the point estimate and the lower and upper limit of the confidence limit of the HIV rate among those actually tested.

## Results

### Audit characteristics

There were 49 audits from 23 centres representing 7037 persons and the median period of time for retrospective data collection was 1.5 years. Of the audits conducted, 11 (22.4%) were from each of Southern and Central Europe, 13 (26.5%) from Eastern, and 14 (28.6%) from Northern Europe. There were 16 audits (32.7%) for TB (representing 1401 persons), 9 (18.4%, 1274 persons) for NHL, 5 for anal cancer (10.2%, 531 persons), 6 for cervical cancer (12.2%, 583 persons), 10 for hepatitis (20.4%, 2681 persons) and 3 (6.1%, 567 persons) for oesophageal candidiasis. All regions performed audits on all ICs, with the exception of oesophageal candidiasis, with neither Southern nor Central Europe being represented. A summary of the audits is shown in Table 1. The median number of audits per site was 3 (IQR 2–4), with a median number of 57 patients/year seen with a specific IC and not known to be HIV+ (IQR 20–140).

**Table 1:** Summary of Audit Results.

Region	All		South		Central		North		East	
Audits (number, %)	49	100	11	22.4	11	22.4	14	28.6	13	26.5
	Median	IQR	Median	IQR	Median	IQR	Median	IQR	Median	IQR
Audit period (yr)	1.5	1.0–2.3	1.5	1.0–2.5	1.0	1.0–2.0	2.1	1.0–4.6	1.0	1.0–1.7
N Audits	3	2–4	3	2–4	3	2–3	2	1–5	3	3–4
N HIV-/yr	57	20–140	33	20–78	17	11–58	45	12–155	128	62–344
Offer rate^1^	86	60–100	77	26–98	86	72–91	69	33–70	100	97–100
Uptake rate^2^	100	100–100	100	99–100	100	100–100	100	98–100	100	100–100
Test rate	72	32–97	68	21–98	78	30–91	44	22–68	99	86–100
HIV+ rate per1000?	0.9	0.0–4.9	2.9	0.9–6.5	0.0	0.0–4.8	0.4	0.0–5.0	1.2	0.3–2.0
>0.1% HIV+*^3^	29	60.4	8	80.0	4	36.4	7	50.0	10	76.9

^1^calculated for 41 audits.

^2^calculated for 40 audits.

^3^calculated for 48 audits; one centre reported doing no tests and therefore no valid denominator

### Testing rates per audit per region and per IC

The test rate was 72% overall (IQR 32–97), with the lowest rate in Northern Europe (median 44%, IQR 22–68%) and the highest in Eastern Europe (median 99%, IQR 86–100%). A high test rate was defined as >72% of those seen with unknown HIV status being tested for HIV. After adjustment for region, IC and the number of persons per audit, those from Northern Europe were less likely to have a test rate >72% compared to those from Southern Europe (adjusted odds ratio [aOR] 0.12; 95% CI 0.011–1.31, p = 0.082), and there was a weak, non-significant association with number of patients seen per year. Compared to TB and oesophageal candidiasis, those testing for NHL, anal and cervical cancer were less likely to have a high test rate (aOR 0.078; 95% CI 0.0079–0.68, p = 0.021).

### Test offer and uptake rates

Offer rates (number of patients with IC offered an HIV test) were available for 41/49 audits. The offer rate for HIV testing was 86% overall (IQR 60–100%), with the lowest offer rate in Northern Europe (median 69%, IQR 33–70) and the highest in Eastern Europe (median 100%, IQR 97–100%). A high offer rate was defined as a rate of >86%. After adjustment, those from northern Europe were less likely to have a high offer rate than those in Southern Europe (aOR 0.19; 95% CI 0.032–1.09, p = 0.062), although this was marginally statistically significant, possibly due to the small sample size. There were no other regional differences and no association with number of HIV- persons seen per year (p-value 0.80). Those presenting with cancer (NHL, anal or cervical) were less likely to have a high offer of testing compared to those presenting with TB or oesophageal candidiasis (aOR 0.25; 95% CI 0.030–0.77, p = 0.016); no differences were seen comparing TB/oesophageal candidiasis and hepatitis (p = 0.55).

The uptake of an offer of an HIV test was close to complete in all audits (95–100%) across all regions and all IC audits, meaning that nearly all of those who were offered an HIV test accepted to be tested.

### HIV positivity rate per audit per region and per IC

**Fig 1:**
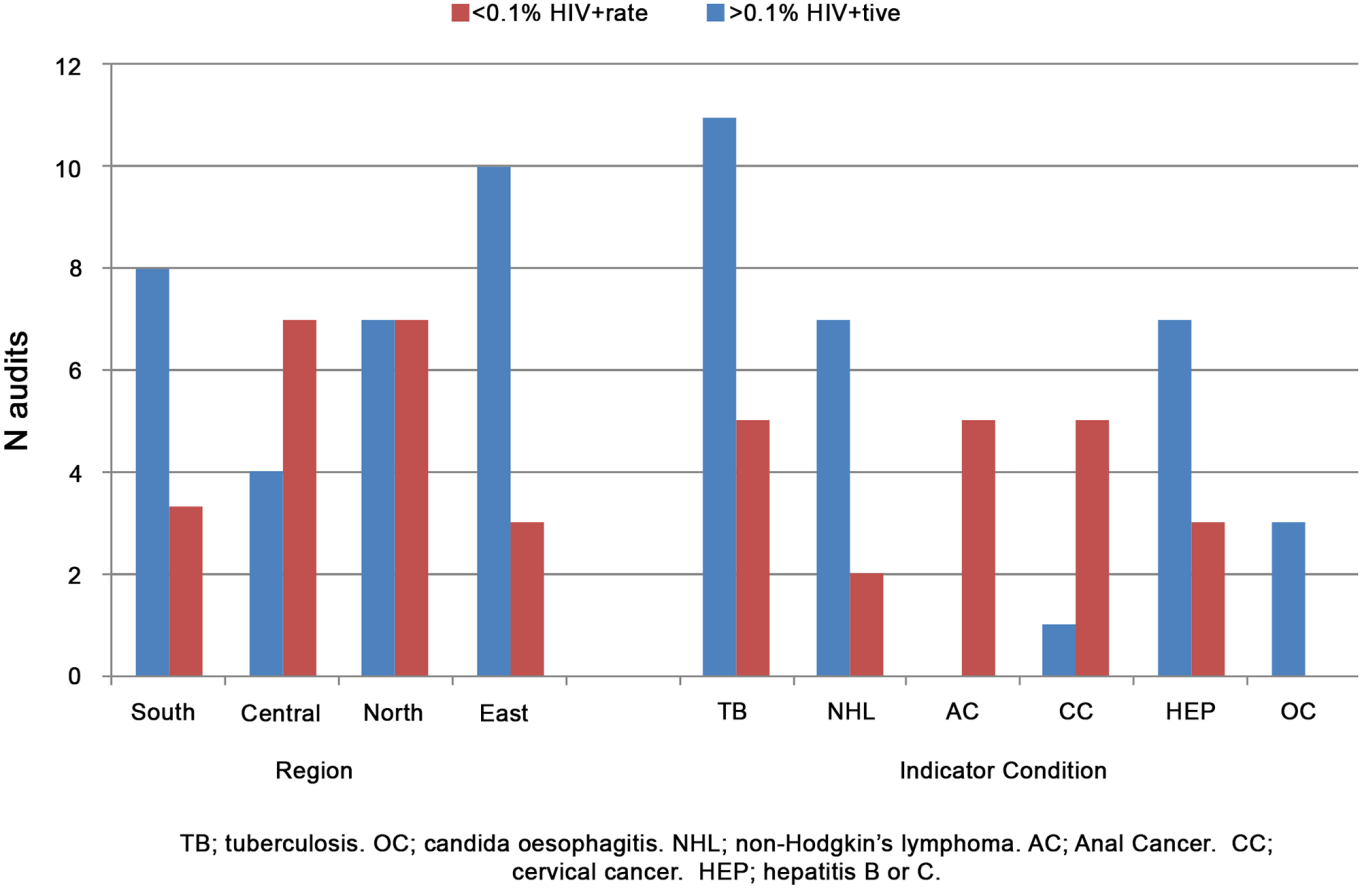
Proportion with high HIV+ rate (>0.1%) across regions and ICs. The figure shows the audits with HIV positive rate < and >0.1% by region and by Indicator Condition (IC).

The median HIV+ rate was 0.9% (IQR 0.0–4.9) (Fig 1) and was highest in Southern Europe (2.9%) and Eastern Europe (1.2%). There was some variation in the proportions with a high positive test rate (defined as >0.1%) across regions and across ICs, shown in Fig 1. Overall, 60.4% (29/48) audits had a positive rate >0.1%, ranging from 80.0% (8/10 audits) in Southern Europe to 4/11 (36.4%) in Central Europe. All (3/3,100%) audits for oesophageal candidiasis had a positive rate >0.1%.

After adjustment for number of patients, region and IC, compared to Southern Europe, those from Central and North were less likely to have a high positive rate of >0.1% (aOR 0.30; 95% CI 0.078–1.14, p = 0.077 and 0.26; 95% CI 0.080–0.85, p = 0.026)), while those clinics testing more patients were more likely to have a high positive rate (aOR 1.57/100 extra patients seen; 95% CI 1.08–2.29, p = 0.019). Compared to TB and oesophageal candidiasis, those testing for NHL, anal or cervical cancer were significantly less likely to have a high positive rate (aOR 0.21; 95% CI 0.48–0.89, p = 0.034), with no differences between TB/oesophageal candidiasis and hepatitis (p = 0.39).

### Potential missed HIV diagnoses

The number testing HIV+ was 113, and we estimate that 105 additional persons would have tested HIV+ if the HIV+ rate within each IC and region was applied to all the persons represented by the audit. The HIV + rate within each IC and region was applied to all the persons represented by the audit, not just those tested, to estimate the number of HIV+ diagnoses potentially missed, together with how many would be missed if the HIV+ rate for each IC/region was as low as the lower 95% CL or as high as the 95% CL, as shown in Table 2.

**Table 2:** Potential Missed HIV Diagnoses, Including Upper and Lower Range.

	Total	South	Central	North	East
Tuberculosis	8 (0, 50)	1 (0, 15)	1 (0, 8)	0 (0, 7)	5 (0, 20)
Non-Hodgkin lymphoma	52 (29, 84)	41 (29, 53)	0 (0, 4)	11 (0, 24)	0 (0, 3)
Anal cancer	0	0	0	0	0
Cervical cancer	0 (0, 2)	0	0	0	0 (0, 2)
Hepatitis B or C	4 (0, 31)	1 (0, 5)	0	3 (0, 10)	0 (0, 16)
Oesophageal candidiasis	41 (28, 59)	0	0	41 (28, 53)	0 (0, 6)
**Total**	**105 (57, 226)**	**43 (29, 73)**	**1 (0, 12)**	**56 (28, 94)**	**5 (0, 47)**

The HIV positivity rate within each IC and region was applied to all the persons represented by the audits, not just those tested, to estimate the number of HIV positive diagnoses potentially missed. The range of the total number missed is shown in parentheses.

Overall, if the rates of HIV+ were the same as those reported from the tested patients and applied to all persons covered by the audit, we have potentially missed >100 diagnoses, including 52 for NHL and 41 for OC. The number potentially missed from Southern Europe was 43 and 56 from Northern Europe. The total number of potentially missed HIV diagnoses was either as low as 57 or as many as 226.

## Discussion

The HIDES study group has successfully launched and implemented an audit system of HIV indicator condition guided HIV testing in healthcare settings. The results show that persons presenting to healthcare settings with conditions widely accepted as IC are not routinely HIV tested. This is particularly true in Northern Europe, with potentially serious consequences for some ICs, especially malignancies, where a high number of potential HIV diagnoses may have been missed if the same rates applied in those not tested as those tested. This confirms previous results showing how persons diagnosed late with HIV have in many cases been in contact with the healthcare system prior to their HIV diagnosis [9,14–16].

The consequences of missing HIV diagnoses are many: for the individual health of the patient, the public health by onward transmission risk and the increased costs to the health- care system. HIV testing therefore needs to be integrated into the routine healthcare for ICs in healthcare settings across Europe.

That the uptake of testing is close to complete across regions and across ICs may indicate that the acceptance of HIV testing is high among persons attending healthcare facilities for other reasons. As patient data is not available from these audits, we cannot rule out that some tests were performed without informed consent, increasing the reported test rates. However, in most clinical settings patients do not object to HIV testing, and high rates of acceptance of testing in patients presenting with ICs are plausible [17].

Comparing the test rates and positivity rates shown in this study to the HIV screening of pregnant women [18–27], it is noticeable how easily routine testing has been introduced in this population group. This is perhaps because the test is not only for the health of the mother, but potentially protecting the unborn child from a serious illness. Although information on HIV test rates and positivity rates are scarce in the literature on HIV screening of pregnant women, country prevalence data collected through official national statistics shows that coverage of screening is very high. In many countries screening coverage is >90%, UK, Ireland, Finland, Denmark and Estonia having nearly complete screening coverage [21–25]. There is evidence that coverage in Eastern Europe varies with rates ranging from 2% in Macedonia to 99% in Estonia. Countries like Denmark and the UK reached their high screening coverage in recent years following the introduction of policies recommending routine screening of all pregnant women, showing the effectiveness of good policy and effective implementation [26,27].

Overall, we found 29/49 audits had a positive rate >0.1%, compared to an undiagnosed HIV positivity rate in pregnant women which is often <0.1%, meaning that the strategy of screening in pregnancy is not a cost-effective intervention, although recommended as essential pre-natal care. The positivity rate in several AIDS defining as well as non-AIDS defining conditions in these audits exceeds the cost-effectiveness threshold of 0.1%. We recommend therefore that the observed barriers to introduce routine HIV testing/screening for persons presenting with defined HIV ICs should be addressed and lessons learned from the introduction of routine screening of pregnant women in many countries.

## Limitations

The data presented are the summary aggregate data from the audits. As individual patient-level data were not available, it has not been possible to take patient variation and medical history into account in the analysis. It is possible that clinics have over-reported HIV testing or that significant changes have occurred in the short time since this audit was performed. The comparatively small number of audits performed has resulted in some uncertainty about the number of potentially missed diagnoses. It is also possible that clinicians have targeted the HIV testing offered to those at perceived greatest risk of testing positive, which may mean that our rate of testing positive is overestimated and that the number of missed diagnoses would be smaller than estimated.

## Conclusions

Testing rates in well-established HIV ICs remain surprisingly low in some regions of Europe despite high prevalence rates, reflecting missed opportunities for earlier HIV diagnosis, treatment and care. Offer rates were higher in Southern than in Northern Europe and in TB and oesophageal candidiasis than in those presenting with NHL, anal or cervical cancer. A significant number (>100) of individuals may have had an earlier opportunity for HIV diagnosis. This also assumes that the same number of people had tested positive among those not offered an HIV test.

The high positivity rate observed through the HIDES study in both AIDS defining and non AIDS defining ICs strongly reinforces the strategy of IC-guided HIV testing, both from the individual and public health perspective. It is recommended that regular auditing of HIV testing in ICs is introduced across Europe. The system developed by HIV in Europe can be easily applied in national and European contexts (www.hiveurope.eu).

## Supporting Information

S1 Table (PDF)
