# Supplementary material for: Auditing HIV Testing Rates across Europe: Results from the HIDES 2 Study
Source: PLoS One. 2015 Nov 11;10(11):e0140845. doi: 10.1371/journal.pone.0140845 (PMC4641587; doi:10.1371/journal.pone.0140845)
Supplement: S1 Table — (PDF) [file pone.0140845.s001.pdf]

Auditing HIV Testing Rates across Europe

Centercode	what_indicatordisease*	not_known_hiv_pos	not_known_hiv_pos_datefrom	not_known_hiv_pos_dateto	number_offered_hivtest	number_hiv_tested	number_hiv_positive	completed_date
4512	1	47	2011-01-01	2012-12-31	36	34	0	2013-05-10
4512	1	30	2009-01-01	2010-12-31	30	30	1	2013-05-10
4512	1	40	2007-01-01	2008-12-31	39	39	1	2013-05-10
1000	1	126	2010-01-01	2011-12-31	115	115	5	2012-04-18
1000	2	22	2010-01-01	2011-12-31	19	19	3	2012-04-18
1000	4	30	2010-01-01	2011-12-31	9	9	0	2012-09-21
1515	6	3	2012-01-01	2012-12-31	1	1	1	2013-05-16
2000	1	191	2007-08-01	2011-12-22	134	130	0	2013-05-07
2000	2	92	2008-07-30	2013-01-25	64	63	10	2013-05-07
2000	3	23	2001-01-01	2011-10-30	5	5	0	2013-05-07
2000	4	43	2007-04-02	2011-10-30	2	2	0	2013-05-07
2000	5	200	2010-11-02	2012-02-16	138	135	1	2013-05-07
2003	2	281	2009-01-01	2009-12-31		91	3	2013-04-15
2003	5	297	2012-01-02	2012-12-31	297	297	0	2013-04-16
	5	185	2013-01-01	2013-03-30	100	100	3	2013-09-23
2019	3	167	1998-07-09	2012-10-25		2	0	2013-04-24
2019	6	524	1997-11-13	2012-10-02		90	8	2013-04-24
2023	1	188	2011-01-10	2012-09-18	162	161	8	2013-05-13
2031	1	121	2010-01-04	2012-08-01	41	40	0	2012-12-18
2519	1	12	2012-01-01	2012-12-31	11	11	0	2013-05-10
2519	2	1	2012-01-01	2012-12-31	1	1	1	2013-05-10
2519	5	23	2012-01-01	2012-12-31	20	20	0	2013-05-10
2522	1	29	2012-01-01	2012-12-31	21	21	1	2013-05-21
2522	2	58	2012-01-01	2012-12-31	45	45	0	2013-06-04
2522	5	59	2012-01-01	2012-12-31	35	35	0	2013-05-21
4509	1	50	2010-12-31	2012-06-30	43	43	2	2013-05-15
	1	62	2012-01-01	2012-12-31	62	62	4	2013-05-03
6514	1	38	2011-01-01	2011-12-31	26	26	2	2012-10-19
6514	2	78	2011-01-01	2011-12-31	73	2	1	2013-03-13
6514	3	4	2011-01-01	2011-12-31	0	0	0	2012-10-19
6514	4	24	2011-01-01	2011-12-31	5	5	0	2013-03-06
7001	2	419	2010-01-01	2012-10-25	109	109	1	2012-10-25
7001	5	395	2010-01-01	2012-10-25	237	234	2	2012-10-25
8000	1	143	2009-01-02	2009-12-23	100	100	0	2012-10-08
9002	3	18	2010-01-01	2013-05-01		5	0	2013-05-19
9002	4	40	2010-12-31	2013-05-01		5	0	2013-05-13
9100	5	419	2011-01-01	2011-12-31	408	405	3	2013-04-22
9101	3	319	2011-01-01	2011-12-31	267	267	0	2013-04-29
9101	4	343	2011-01-01	2011-12-31	321	320	1	2013-04-29
9101	5	412	2011-01-01	2011-12-31		408	3	2012-11-17
9501	1	114	2011-01-01	2012-12-31		19	1	2013-04-23
9805	2	128	2012-01-01	2012-12-31		80	0	2013-05-04
9806	2	195	2009-12-01	2012-09-14	195	167	2	2012-09-14
9806	5	587	2009-12-01	2012-09-02	587	587	9	2012-09-02
9806	6	40	2010-12-31	2012-07-01	40	40	13	2013-04-19
9810	4	103	2011-09-23	2010-01-22	103	103	0	2012-08-27
9810	1	100	2009-09-23	2009-10-28	100	100	2	2012-08-01
9810	1	110	2011-01-28	2011-12-28	110	110	19	2012-08-03
9810	5	104	2010-01-27	2011-09-30	104	104	2	2012-08-27

* Indicatordisease	
1	A) Tuberculosis
2	B) Non-Hodgkin's lymphoma
3	C) Anal cancer
4	D) Cervical cancer
5	E) Hepatitis B and C
6	F) Candida esophagitis
